# Mechanistic Fingerprinting Reveals Kinetic Signatures of Resistance to Daptomycin and Host Defense Peptides in *Streptococcus mitis-oralis*

**DOI:** 10.3390/antibiotics10040404

**Published:** 2021-04-08

**Authors:** Michael R. Yeaman, Liana C. Chan, Nagendra N. Mishra, Arnold S. Bayer

**Affiliations:** 1 Division of Molecular Medicine, Department of Medicine, Los Angeles County Harbor-UCLA Medical Center, Torrance, CA 90502, USA; MRYeaman@ucla.edu (M.R.Y.); lchan@lundquist.org (L.C.C.); 2 Division of Infectious Diseases, Department of Medicine, Los Angeles County Harbor-UCLA Medical Center, Torrance, CA 90502, USA; nmishra@lundquist.org; 3 The Lundquist Institute for Biomedical Innovation at Harbor-UCLA Medical Center, Torrance, CA 90502, USA; 4 Department of Medicine, David Geffen School of Medicine, University of California (UCLA), Los Angeles, CA 90024, USA

**Keywords:** *Viridans streptococci*, daptomycin resistance, host defense peptides

## Abstract

*Streptococcus mitis-oralis (S. mitis-oralis)* infections are increasingly prevalent in specific populations, including neutropenic cancer and endocarditis patients. *S. mitis-oralis* strains have a propensity to evolve rapid, high-level and durable resistance to daptomycin (DAP-R) in vitro and in vivo, although the mechanism(s) involved remain incompletely defined. We examined mechanisms of DAP-R versus cross-resistance to cationic host defense peptides (HDPs), using an isogenic *S. mitis-oralis* strain-pair: (i) DAP-susceptible (DAP-S) parental 351-WT (DAP MIC = 0.5 µg/mL), and its (ii) DAP-R variant 351-D10 (DAP MIC > 256 µg/mL). DAP binding was quantified by flow cytometry, in-parallel with temporal (1–4 h) killing by either DAP or comparative prototypic cationic HDPs (hNP-1; LL-37). Multicolor flow cytometry was used to determine kinetic cell responses associated with resistance or susceptibility to these molecules. While overall DAP binding was similar between strains, a significant subpopulation of 351-D10 cells hyper-accumulated DAP (>2–4-fold vs. 351-WT). Further, both DAP and hNP-1 induced cell membrane (CM) hyper-polarization in 351-WT, corresponding to significantly greater temporal DAP-killing (vs. 351-D10). No strain-specific differences in CM permeabilization, lipid turnover or regulated cell death were observed post-exposure to DAP, hNP-1 or LL-37. Thus, the adaptive energetics of the CM appear coupled to the outcomes of interactions of S. *mitis-oralis* with DAP and selected HDPs. In contrast, altered CM permeabilization, proposed as a major mechanism of action of both DAP and HDPs, did not differentiate DAP-S vs. DAP-R phenotypes in this *S. mitis-oralis* strain-pair.

## 1. Introduction

The *Streptococcus mitis-oralis* (*S. mitis-oralis*) subgroup of viridans group streptococci (VGS) includes *S. mitis*, *S. oralis*, *S. gordonii* and *S. parasanguinis* [1,2,3,4]. This subgroup, especially *S. mitis*, is an important and emerging cause of serious community- and hospital-acquired infections, including infective endocarditis and sepsis syndromes in neutropenic cancer patients (i.e., “toxic Strep shock syndrome”) [1,2,3,4,5,6,7,8]. A major, but often overlooked, problem associated with *S. mitis-oralis* infections is the emergence of antimicrobial resistance. *S. mitis-oralis* strains are frequently resistant in vitro to penicillin (~20–40% of isolates) and cephalosporin antibiotics, including third-generation agents such as ceftriaxone (~10–25% of isolates) [8,9,10,11]. Moreover, *S. mitis-oralis* strains can be vancomycin-tolerant [12]. These issues have raised the notion of a first-line role for lipopeptides, including daptomycin (DAP), for the treatment of such infections. However, a substantial proportion of clinically derived *S. mitis-oralis* strains can rapidly evolve high-level and durable DAP resistance phenotypes (DAP-R; MIC > 256 µg/mL) in vitro, ex vivo and in vivo after DAP exposure [13,14]. For example, Garcia-de-la-Maria et al. [13] reported that >25% of *S. mitis-oralis* strains from bacteremic patients developed high-level and durable DAP-R after serial exposure to this agent in vitro (MICs > 256 µg/mL). A similar DAP-R phenomenon was seen following exposure to DAP alone in simulated cardiac endocarditis vegetations ex vivo [14] and in vivo within vegetations from experimental animals with endocarditis initially infected with a DAP-S parental strain [13]. Such extremely high-level DAP-R phenotypes have only rarely been observed among DAP-R *S. aureus* or enterococci [15,16,17,18].

Although DAP-R VGS infections have been infrequently clinically documented to date, the extensive prophylactic and therapeutic use of DAP, plus a potential for DAP MIC ‘creep’ (as has been observed in enterococci) [17,18], are worrisome. Thus, understanding the mechanism(s) of DAP-R in *S. mitis-oralis* strains is of great significance for this emerging pathogen.

Recently, we reported that DAP-R in *S. mitis-oralis* involves unique mechanisms not previously documented in *S. aureus* and enterococci. These include: (i) major perturbations in cell membrane (CM) phospholipid repertoires, featuring the essential repression of two lipid species critical to DAP CM binding and oligomerization (phosphotidylglycerol and cardiolipin) [19], (ii) loss-of-function mutations in genes involved in biosynthesis of these phospholipids [19,20,21], (iii) corresponding shifts in metabolomic profiles of DAP-R strains, particularly involving glycolytic pathway intermediates [22] and (iv) altered DAP–CM interaction dynamics, characterized by enhanced ability to hyper-accumulate DAP in specific cells within a given streptococcal chain [19,21].

Although the above studies have been crucial for understanding the genetic, phenotypic and metabolic underpinnings of DAP-R in *S*. *mitis-oralis* strains, they did not address either the kinetics or integrative mechanistic responses of the organism during DAP exposures. Thus, we employed a recently developed flow cytometric fingerprinting technique to disclose kinetic signatures corresponding to DAP-R in *S. mitis-oralis*. This unique methodology allowed us to simultaneously examine multiple, distinct responses to DAP, and compare these adaptations with other cationic molecules (HDPs) in an isogenic, DAP-S vs. DAP-R *S. mitis-oralis* strain-pair.

## 2. Materials and Methods

### 2.1. S. mitis-oralis Strains

As previously detailed [13], the DAP-S parental 351-WT *S.* *mitis-oralis* strain was a clinical bacteremic isolate from a patient with endocarditis (kindly supplied by Dr. Jose Miro; Barcelona, Spain). This strain was identified as *S. mitis* by microbiologic-biochemical assays, and *S. mitis-oralis* by MALDI-TOF (Matrix-assisted laser desorption/ionization–time-of-flight); of interest, this strain’s genomic sequencing was closer to *S. oralis* than *S. mitis* [19]. For ease of discussion, we have designated this strain as *S. mitis-oralis*. The DAP-R variant (351-D10) was previously selected by serial in vitro passage for 10 days in DAP (20 µg/mL) as described [19], and the DAP-R phenotype in this variant was stable after 5 days of passage in antibiotic-free media [19]. For this investigation, the *S. mitis-oralis* strains were cultured in brain heart infusion broth (BHI) (Becton-Dickinson) at 37 °C overnight without shaking, pelleted by centrifugation, washed in PBS (phosphate-buffered saline), and resuspended to the appropriate CFU (colony-forming units) (culture-confirmed) by spectrophotometry for each specific assay. To achieve adequate log-phase cells for these assays, overnight culture preparations were then incubated in large-volume (500 mL) cultures of fresh BHI (without shaking) for an additional 3 h at 37 °C.

Susceptibilities of the study strains to DAP (previously reported) were determined using a microbroth dilution assay per recommendations of the Clinical Laboratory Standards Institute (CLSI) in calcium-supplemented (Ca^2+^) Mueller-Hinton broth [19,20,21,22,23]. The DAP-S parental strain 351-WT has a DAP MIC of 0.5 µg/mL, while its isogenic DAP-R variant 351-D10 has an MIC > 256 µg/mL. The MIC determinations were performed a minimum of two times in independent experiments conducted on separate days.

### 2.2. Host Defense Peptides (HDPs)

Two cationic peptides important in the mammalian innate host response were studied. Human neutrophil peptide-1 (hNP-1; Peptides International, Louisville, KY, USA) is a prototypic alpha-defensin, naturally found in human neutrophil specific granules, which is important in intra-phagolysosomal killing of bacterial pathogens [24]. Also, human LL-37 (Peptides International, Louisville, KY, USA) is an endogenous cathelicidin HDP (host defense peptide) elaborated by both epithelial tissues and neutrophils [25]. Each of these peptides exhibits an intrinsic net cationic charge and has been confirmed to exert antimicrobial activity against Gram-positive bacterial pathogens in vitro [19,26,27].

### 2.3. Susceptibility to DAP and HDPs by Time-Kill Assays

Susceptibilities of the study strain-pair to HDPs were determined using a solution phase method as previously detailed [19,26]. In brief, washed organisms were inoculated into piperazine-N, N′-bis (2-ethanesulfonic acid) (PIPES) buffer (10 mM) and adjusted to pH 7.5 [28]. The buffer was inoculated with 10^6^ CFU/mL of a given logarithmic phase organism and incubated with each peptide at a final concentration of 10 µg/mL for 1, 2 or 4 h at 37 °C. This concentration was determined by extensive pilot studies to exert measurable but incomplete killing of the parental DAP-S strain over the 4 h incubation time-period. After incubation, the suspensions were quantitatively cultured in duplicate onto Mueller-Hinton agar plates. Colonies were enumerated after incubation for a minimum of 24 h at 37 °C, and the mean percent killing of the initial inoculum was calculated. All antimicrobial assays were performed a minimum of two times in independent studies on separate days, and data were analyzed for statistically significant outcomes.

Killing of the *S. mitis-oralis* strain-pair by DAP over a similar 4 h time-period was carried out, using the same final inoculum of logarithmic phase cells, exposed to 10 µg/mL DAP (with calcium supplementation as above). This concentration of DAP was also sublethal for the parental DAP-S strain at the above inoculum over a 4 h incubation period in pilot studies. As above for HDPs, DAP killing assays were performed a minimum of two times in independent studies on separate days and mean percent killing data were analyzed for statistically significant outcomes.

In addition to untreated growth controls in MHB, the following non-specific CM-active ‘detergent’ controls were used in these killing assays: sodium dodecyl sulfate (SDS (10% wt/vol; Ambion)) and 70% ethanol (ETOH). Pilot studies confirmed these latter two agents were rapidly bactericidal against this *S. mitis-oralis* strain-pair (data not shown).

### 2.4. BODIPY-DAP Binding

A fluorescent-conjugated version of DAP (BODIPY-DAP; 4,4-difluoro-4-bora-3a,4a-diaza-s-indacene–DAP) was used to quantify DAP-binding interactions with the *S. mitis-oralis* study strains. BODIPY-DAP is fluorescent due to DAP being covalently conjugated with the BODIPY moiety. As with the parental DAP, BODIPY-DAP requires calcium for activation of its antibacterial activity. Based on extensive pilot data with the two *S. mitis-oralis* study strains (not shown) and prior publications [29,30], BODIPY-DAP retained its antimicrobial activity equivalent to native DAP in vitro. BODIPY-DAP was prepared and obtained from Dr. Warren Rose (University of Wisconsin School of Pharmacy, Madison, WI, USA). Excitation and emission wavelengths used for the BODIPY-DAP flow cytometry studies were 488 nm (argon laser) and 525 ± 15 nm (band pass detector), respectively (FACSCalibur; Becton-Dickinson Biosciences, San Jose, CA, USA).

For comparative binding studies by flow cytometry, *S. mitis-oralis* strains (10^6^ CFU/mL) were exposed to BODIPY-DAP (128 µg/mL) in phosphate-buffered saline, 10% Mueller-Hinton broth (MHB; Becton-Dickinson, San Jose, CA, USA) or Roswell Park Memorial Institute (RPMI) medium (Fischer Scientific, Hampton, NH, USA) with Ca^+2^ supplementation (50 ug/mL) at pH 7.5 for 15 min at 37 °C. These three distinct media conditions represent: physiologic ionicity, enriched artificial media or a microenvironment mimicking host cell growth, respectively. After incubation, samples were washed to remove unbound BODIPY-DAP and processed for flow cytometry. Cells were gated based on mean channel fluorescence decades: 0–10^1^ = low binding, 10^1^–10^2^ high binding and >10^2^ hyper-accumulating. The percentage of cells with these three population groups was quantified under each experimental condition for each strain. Data were normalized to unlabeled cells processed and evaluated identically.

### 2.5. Kinetic Signatures by Multi-Parameter Flow Cytometry

We employed six-parameter multicolor flow cytometry as previously detailed [28] to analyze the following specific physiologic responses: (i) change in cell size and/or surface area (e.g., osmostasis, forward scatter (FSC)), (ii) cytoplasmic refractivity (nucleic acid condensation, side scatter (SSC)), (iii) perturbation of CM energetics (polarization/transmembrane potential energetics (Δψ), (ENR), FL-1), (iv) loss of CM integrity (e.g., permeability (PRM), FL-2), (v) CM/phospholipid bilayer turnover (phosphatidyl serine accessibility (PSA), FL-4) and (vi) regulated cell death (caspase-like protease induction (CSP), FL-1).

The following fluorophores were used in combination with a FACSCalibur^®^ cytometer (Becton-Dickinson Biosciences, San Jose, CA, USA): 3,3-dipentyloxacarbocyanine (DiOC5; excitation (Ex): 484 nm/emission (Em): 660 nm; Invitrogen, Carlsbad, CA, USA) for ENR, propidium iodide (PI; Ex: 535 nm/Em: 620 nm; Sigma, St. Louis, MO, USA) for PRM, annexin-V allophycocyanin conjugate (ANX-V; Ex: 650 nm/Em: 660 nm, Invitrogen, Carlsbad, CA, USA) for PSA and Cell Event^®^ Caspase-3/7 Green (C-3/7; Ex: 502 nm/Em: 530 nm, Invitrogen, Carlsbad, CA, USA) for CSP. This latter reagent is specifically cleaved only by proteases induced in phylogenetically conserved programmed cell death-like pathways, and is non-fluorescent until such cleavage [28]. Forward scatter (FSC) and SSC were measured in parallel.

Logarithmic-phase organisms were adjusted to 10^8^ CFU/mL in PIPES pH 7.5 and exposed to 10 µg/mL of each HDP-of-interest or DAP for 1–4 h at 37 °C (unlabeled DAP was used in these studies). Based on extensive pilot data, this peptide concentration corresponded to ~50% survival of the *S. mitis-oralis* inoculum. Consistent with the time-killing assays above, sodium dodecyl sulfate (SDS (10% wt/vol; Ambion)) or 70% ethanol (ETOH) served as positive controls for killing, and buffer alone served as a negative control in each experiment. A triple-stain cocktail containing DiOC_5_ (0.5 µM), PI (5.0 µg/mL), and AXN-V (2.5 µL/mL) in 50 mM potassium-containing MEM (K^+^ MEM, without phenol red; Sigma, St. Louis, MO, USA) was added to each sample following incubation. Parallel samples were incubated with 30 µl of C-3/7 reagent for 30 min at 37 °C following peptide exposure. Samples were stained at room temperature for 15 min before flow cytometry. After incubation, 400 mL of buffer was added to each reaction to minimize background signal by dilution. Fluorescence of a minimum of 10,000 cells was acquired from each sample, and results from a minimum of two independent studies conducted on different days were used for statistical analysis.

### 2.6. Statistical Analysis

Student’s *t* test or Mann–Whitney *U* test were applied as appropriate for continuous or discontinuous data to evaluate significant differences. A *p*-value of <0.05 was considered significant.

## 3. Results

### 3.1. BODIPY-DAP Binding Interactions

**Figure 1:**
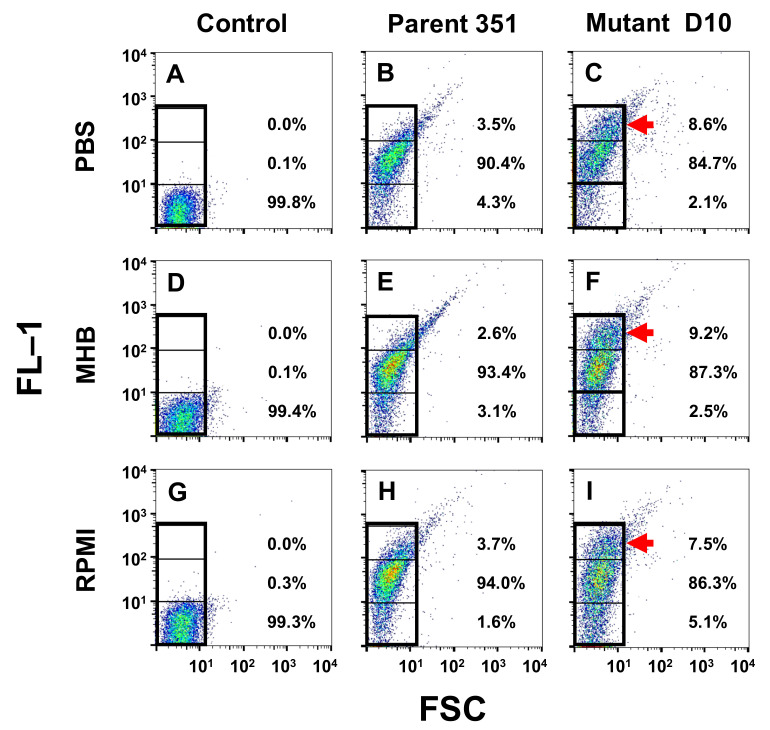
Comparative binding of BODIPY-DAP to DAP-S and 351-D10 DAP-R study strains under distinct conditions in vitro (**A–I**). Exposure of strains to BODIPY-DAP under different conditions (PBS, MHB or RPMI) was performed as detailed in the Methods Section. Percentages of the total cell population meeting FL-1 gating criteria (black boxes) are indicated, corresponding to low-binding (0–10^1^), high-binding (10^1^–10^2^) and hyper-accumulating (>10^2^). Note the substantial 2- to 4-fold increase in hyper-accumulating subpopulations in DAP-R strain D10 (**C**, **F**, **I**; red arrows) vs. the same gating in DAP-S strain 351. Mean channel fluorescence of strain D10 hyper-accumulating subpopulations was 219 ± 48 (PBS), 224 ± 53 (MHB) and 212 ± 39 (RPMI) fluorescence units, as compared to 62 ± 22 (PBS), 53 ± 16 (MHB) and 69 ± 31 for high-binding or 7 ± 2 (PBS), 8 ± 3 (MHB) or 9 ± 3 (RPMI) for low-binding populations, respectively. FSC, forward scatter.

As compared to controls, both strains bound BODIPY-DAP readily: >90% of DAP-S 351-WT cells and >84% of DAP-R 351-D10 cells bound BODIPY-DAP at relative fluorescent units (RFUs) > 10^1^ (Figure 1). Interestingly, despite several distinct media conditions tested (PBS, MHB or RPMI), the DAP-R 351-D10 strain consistently exhibited a substantial sub-population frequency of cells that exhibited high-level binding of BODIPY-DAP (>10^2^ RFUs) ~2–4-fold higher than the DAP-S 351-WT strain (Figure 1C,F,I). It should be noted that for both strains, a “tail” subpopulation of cells extending beyond the gating profile was observed. Because samples were sonicated immediately prior to cytometery, it is unlikely these cells represent clumping. Rather, as supported by prior fluorescent microscopy findings [19], DAP exposures appear to induce chain extension as a result of diminished or failed fission necessary for chain separation.

### 3.2. DAP- or HDP-Mediated Timed-Killing

**Figure 2:**
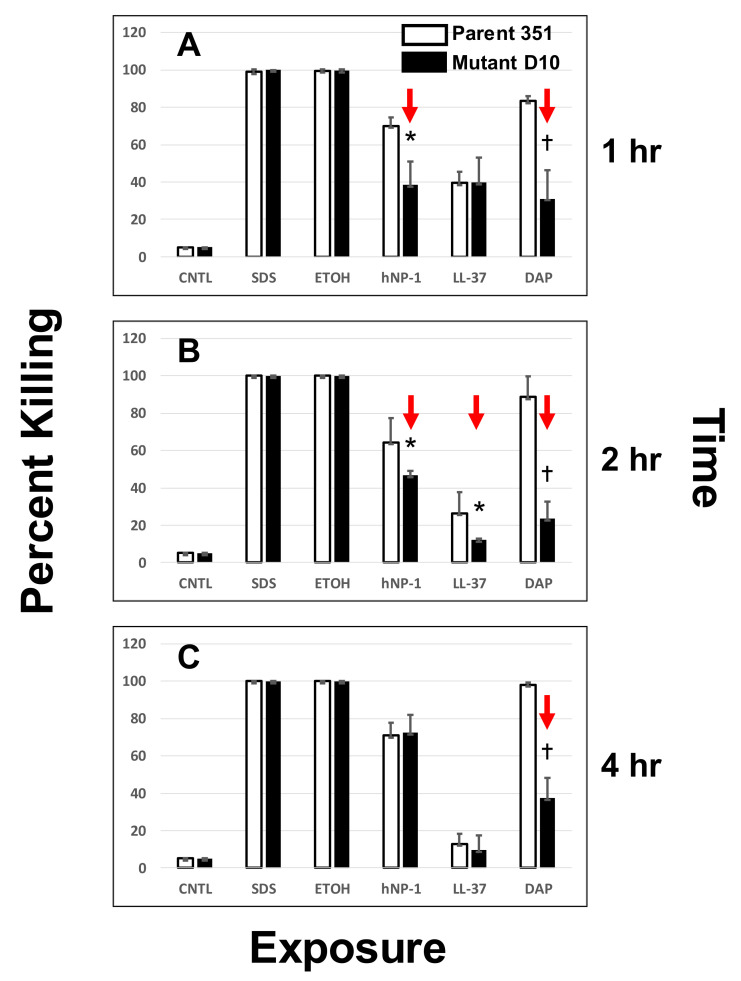
Kinetics of DAP and HDP susceptibilities of study strains DAP-S and 351-D10 DAP-R at 1, 2 or 4 h (panels (**A**), (**B**) or (**C**), respectively) in vitro. Exposure conditions and quantitative culture methods were performed as detailed in the Methods Section. Significant differences in strain susceptibilities (red arrows) represent *p* < 0.01 (†) or *p* < 0.05 (*).

Temporal killing profiles of the *S. mitis-oralis* strain-pair by DAP and two HDPs are shown in Figure 2. At 1 h exposures (Figure 2A), the parental DAP-S strain 351-WT was significantly more susceptible to both DAP and hNP-1 vs. DAP-R strain 351-D10. In contrast, strains 351-WT and 351-D10 were equally susceptible to LL-37 at this time point. At both 2 and 4 h exposure time points (Figure 2B,C), the DAP-S strain 351-WT continued to be significantly more susceptible to DAP killing vs. the DAP-R 351-D10 strain. In contrast, the DAP-S 351-WT strain was significantly more susceptible to hNP-1 at 2 h, but not at 4 h post-exposure to hNP-1. Of note, neither strain was particularly susceptible to LL-37-mediated killing across the 4 h exposure period. As expected, both strains were highly susceptible to rapid killing by the control CM-active microbicidal agents, SDS or ETOH.

### 3.3. Kinetic Signatures by Multi-Parameter Flow Cytometry

**Figure 3:**
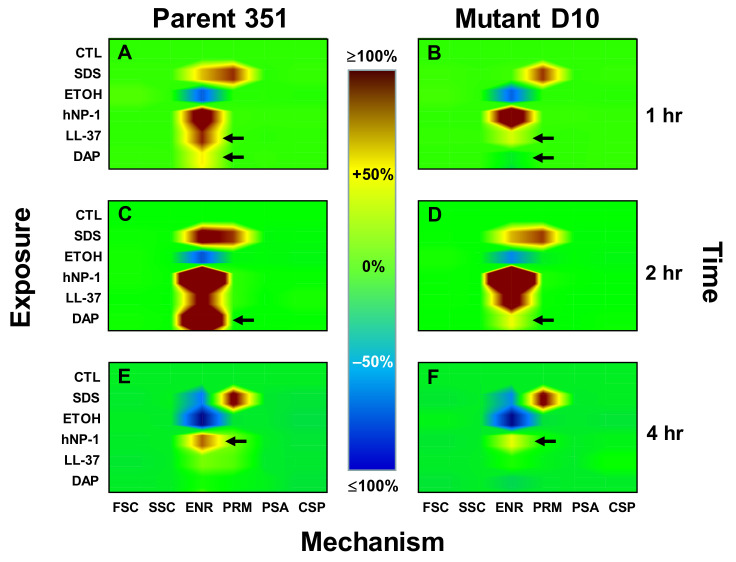
Mechanistic signature mapping of DAP and HDPs against 351 DAP-S and 351-D10 DAP-R study strains in vitro. Exposure conditions (*y* axis) included control (CTL) (buffer alone), the indiscriminant membrane detergent sodium dodecyl sulfate (SDS), hNP-1, LL-37 and DAP, as detailed in the Methods Section. Mechanisms of action (*x* axis) were determined using multicolor flow cytometry: forward scatter (FSC) (cell size/shape), side scatter (SSC) (intracellular refraction indicative of cytoplasm condensation), cellular energetics (ENR) (transmembrane potential), CM permeabilization (PMR) (propidium iodide uptake), negatively charged phospholipid accessibility (PSA) (cytoplasmic membrane turnover (e.g., extracellular exposure of intracellular leaflet bilayer)) and cell death protease activation (CDP). Percent increases (yellow-orange-red) or decreases (blue-indigo-violet) in respective mechanisms of action recorded 1–4 h post exposure are integrated within each mechanistic fingerprinting map panel, each representing >1 million data points derived from a minimum of 3 independent experiments. Figure panels (**A**), (**C**) and (**E**) indicate kinetic response mechanisms in parent strain WT-351 at 1, 2 and 4 hrs, respectively; panels (**B**), (**D**) and (**F**) indicate comparative responses in the mutant 351-D10 strain at the identical timepoints.

Comparative mechanistic kinetics of DAP and the two HDPs were assessed in each of the *S. mitis-oralis* strains, as summarized in Figure 3. Each panel represents results from a minimum of 2.1 million data points derived from a minimum of three independent experiments. The specific mechanisms of action (FSC, SSC, ENR, PRM, PSA, CSP) of DAP relative to prototypic HDPs were compared to SDS or ETOH microbicidal controls at 1, 2 and 4 h to assess kinetic responses to DAP exposure, as detailed below.

#### 3.3.1. One-Hour Exposure Time Point 

In parental strain 351-WT, DAP caused low-level hyperpolarization (ENR +36.5%; *p* < 0.05 vs. untreated control; Figure 3A), while both hNP-1 and LL-37 caused more extensive hyperpolarization of this strain (ENR +139% and 77%, respectively; *p* < 0.01; Figure 3A). Neither DAP, hNP-1 nor LL-37 induced detectable changes in FSC, SSC, PRM, PSA or CSP in strain 351-WT. By comparison, neither DAP nor LL-37 substantially changed ENR in strain 351-D10 (Figure 3B). However, hNP-1 exerted a strong hyperpolarization effect on this latter strain, similar to that seen in strain 351-WT at this time point (ENR +146%; *p* < 0.001 vs. control) (Figure 3B). As expected, SDS caused equivalent and moderate increases in PRM of both strains (PRM +79% (351-WT) and +82% (351-D10); *p <* 0.01 vs. untreated controls). Also, as anticipated, ETOH de-energized both strains at this time point (ENR −54% (351-WT) and −59% (351-D10); *p* < 0.05 vs. untreated control cells). Interestingly, ENR of strain 351-D10 was unaffected by SDS at 1 h exposure, in distinction from strain 351-WT (Figure 3B).

#### 3.3.2. Two-Hour Exposure Time Point 

In parental strain 351-WT, DAP caused significantly greater hyperpolarization at this time point (ENR +246%; *p* < 0.001) than seen at 1 h (Figure 3C vs. Figure 3A). However, DAP induced only a minimal impact on ENR (+16%; non-significant [NS]) in strain 351-D10 after 2 h exposure (*p* < 0.001; Figure 3C vs. Figure 3D). hNP-1 significantly and equivalently increased polarization in both strains, 351-WT and 351-D10, by this time point (ENR +219% and +221%; NS). ENR was significantly increased in both strains vs. the 1 h time point (*p* < 0.001; Figure 3A–D). Likewise, LL-37 generated equivalent and modest increases in ENR in both strains by 2 h (ENR +152% (351-WT) and +149% (351-D10); NS; Figure 3C, D). Notably, while ETOH modestly and equivalently de-energized both strains at 2 h (ENR; −48% (351-WT) and −55% (351-D10); NS), SDS increased ENR in both strains relative to respective outcomes at 1 h (ENR +96% and 41%; *p* < 0.05; Figure 3C,D). Similarly, as anticipated, SDS increased PRM in both strains vs. untreated controls at 2 h (not significantly different from the 1 h time point) (Figure 3A–D). None of the agents tested induced detectable changes in FSC, SSC, PSA or CSP.

#### 3.3.3. Four-Hour Exposure Time Point 

By 4 h exposure, both the parental 351-WT and 351-D10 strains were able to normalize their CM energetics equivalent to that of untreated controls during both DAP and LL-37 exposures (NS; Figure 3E,F). A significant difference was observed in ENR between strains exposed to hNP-1 at the 4 h time point, the parental 351 strain remained modestly hyperpolarized (ENR +66%), while the 351-D10 strain was only minimally hyperpolarized (ENR +21%; *p* < 0.05). Both strains were equivalently de-energized by ETOH and permeabilized by SDS at this time point (Figure 3E,F). As for the other time points above, no detectable changes in FSC, SSC, PSA or CSP were observed at 4 h exposure in either strain.

## 4. Discussion

A number of phenotypic, metabolic and genotypic analyses have studied potential mechanisms involved in phenotypic DAP-R in *S. mitis-oralis*, most of which have utilized the same DAP-S/DAP-R strain pair described above [19,20,21,22,23,29,31,32,33]. These studies identified potential mechanistic themes relevant to the current findings. For example, in previous fluorescent microscopy investigations [19], it was demonstrated that among streptococcal chains of DAP-R 351-D10 (but not DAP-S 351-WT) *S. mitis-oralis* cells, only a few individual cells hyper-accumulate DAP, while the remaining cells bound little or no DAP. In contrast, individual DAP-S parental cells uniformly accumulated DAP within a given chain at modest, but equivalent levels [19]. These data suggested that DAP hyper-accumulation in DAP-R strains may be a functional specialization in which specific cells ‘altruistically’ protect other cells in the greater bacterial community from microbicidal levels of DAP exposure [19]. This DAP hyper-accumulation phenotype does not occur universally in all DAP-R strains, suggesting that it may be a strain-specific mechanism of DAP-R [29].

Our current flow cytometry studies support the above seminal observations [19]. Thus, under several distinct microenvironmental growth conditions, a specific but important subpopulation of DAP-R *S. mitis-oralis* cells rapidly bound high levels of DAP to a logarithmically greater extent than DAP-S parental cells. These data are the first to quantitate the extent and temporal kinetics of this selective hyper-accumulation phenomenon in DAP-R strains. Investigations in-progress are pursuing single-cell genomic analytics of such DAP hyper-accumulating vs. non-hyper-accumulating cells to define the comparative genetic determinants of these distinct microbial phenotypes [30,34].

The present multiparameter flow cytometric fingerprinting studies demonstrated the specific kinetics and mechanisms of killing in DAP-S vs. DAP-R *S. mitis-oralis* strains vs. a cadre of diverse cationic peptides. This technique provided: (i) plausible explanations for differential adaptive mechanisms following exposures to DAP and HDPs, and (ii) time-dependent and mechanistic network comparisons of DAP vs. HDP exposure outcomes. For example, the DAP-S strain was more rapidly and extensively hyper-polarized by DAP, while the DAP-R variant exhibited only a minimal increase in ENR profiles. This result appears to be due to an active, adaptive response of the organism to modulate CM hyperpolarization, and correlates well with the temporal circumvention of killing by the DAP-R variant. In turn, these data support the notion that the net state of CM energetics and transmembrane potential (ΔΨ) are primary DAP-adaptive drivers of differential susceptibility phenotypes in this strain-set. The mechanistic underpinning(s) of such adaptive CM re-equilibration corresponding to resistance is unclear. Of note, CM potential (ΔΨ) is critical to the distribution of cell division proteins, as well as to ultimate bacterial cell fission, and maintaining and restoring dysregulated CM polarization and energetics are key roles of proteins, such as MinD [35,36]. Importantly, in Gram-positive bacteria, MinD-family proteins accumulate at cell division sites that are also preferentially targeted by DAP [36,37]. Thus, it is plausible that CM repair mechanisms are targeted to those sites most vulnerable to cationic peptide-induced CM injury.

In contrast to DAP, mechanistic profiles observed in response to hNP-1 or LL-37 exposures suggest distinct mechanisms of action and resistance. For example, hNP-1 induced equivalent ENR hyperpolarization in the strain pair at 1 and 2 h time points, but not at 4 h. However, the DAP-R variant was more resistant to killing vs. the DAP-S variant at the 1 and 2 h times relative to 4 h. Thus, microbicidal events occurring early post-exposure (e.g., within 2 h) are likely responsible for ultimate differences in hNP-1 susceptibility phenotypes in this strain pair. Similarly, the DAP-S strain exhibited greater susceptibility to killing by LL-37 than the DAP-R variant at 2 h, but ENR hyperpolarization was similar. These data underscore that (as opposed to DAP), mechanisms of killing by hNP-1 or LL-37 are not primarily dependent on ENR hyperpolarization.

**Figure 4:**
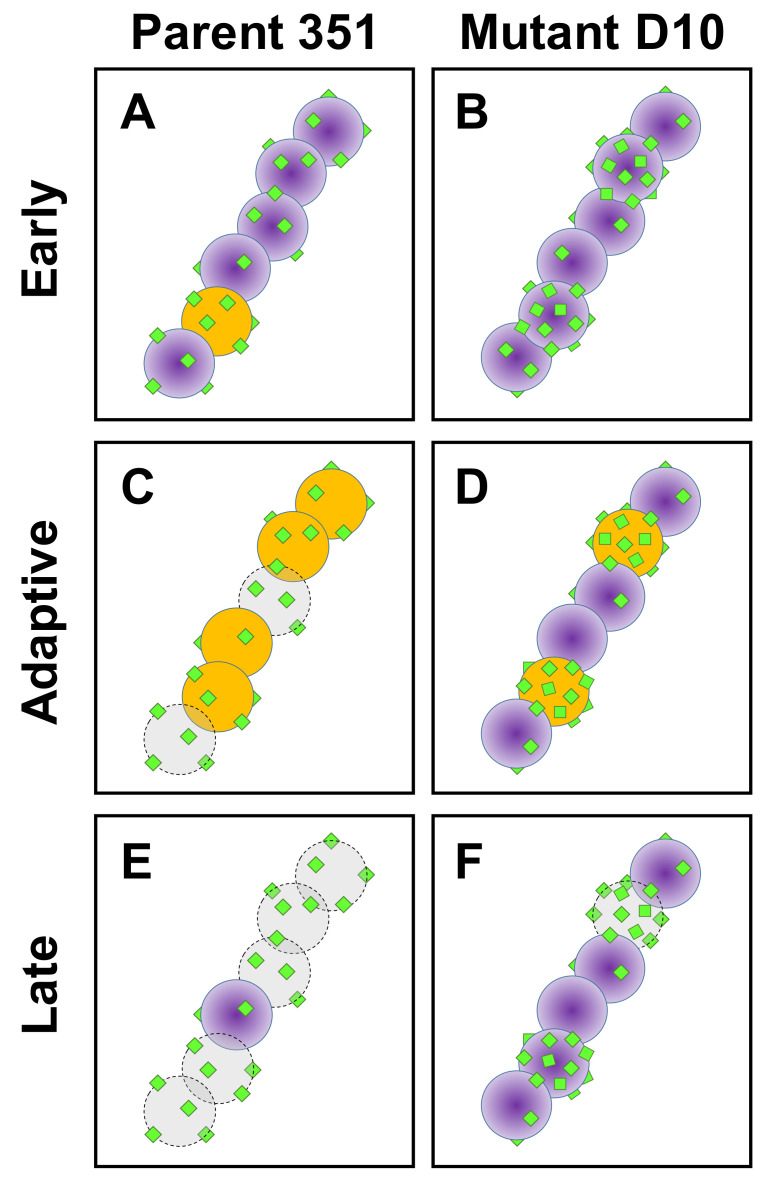
Integrative model of DAP-S vs. DAP-R phenotypes in *S. mitis-oralis* based on our current and prior data. (**A**–**F**) Comparative responses to and outcomes of DAP exposure at different time points in parent and mutant study strains 351 and D10. Early post-DAP exposure, parent 351 (**A**) cells accumulate DAP equivalently and early hyper-polarization is initiated. In contrast, specific cells in DAP-R mutant D10 chains hyper-accumulate DAP (**B**), with minimal to no hyper-polarization. Adaptive responses to restore normal cell membrane energetics fail in parent 351, resulting in hyper-polarization and initial cell death (**C**). By comparison, only specific cells within mutant D10 streptococcal chains appear to hyperpolarize, protecting the remaining population (**D**). Ongoing inability to compensate leads to significant cell death in parent 351 (**E**), whereas mutant D10 organisms have substantially greater survival (**F**). Key: DAP = green squares; viable *S. mitis-oralis* cells = purple; hyperpolarized *S. mitis-oralis* cells = gold; killed *S. mitis-oralis* cells = gray.

Recent metabolomic analyses of the current DAP-S/DAP-R *S. mitis-oralis* strain-pair also divulged interesting distinctions [22]. *S*. *mitis-oralis* strains lack a classical tricarboxylic acid (TCA) cycle, grow preferentially in microaerophilic conditions and adapt their metabolic profile in transitioning from the DAP-S-to-DAP-R phenotype in a manner distinct from DAP-R *S. aureus* [38]. In this respect, the DAP-R 351-D10 strain exhibits delayed glucose catabolism (through glycolysis and lactate dehydrogenase) compared to DAP-S 351-WT [22]. As lactate dehydrogenase plays an important role in maintaining the redox balance, it is highly likely that a dysregulation in redox status contributes to the slower growth of the DAP-R *S. mitis-oralis* strain [22]. These data further support our hypothesis that differential cellular or CM energetics are involved in determining the DAP-R phenotype in *S. mitis-oralis*. Figure 4. Presents a hypothetical sequence of DAP-induced killing of DAP-S vs. DAP-R *S. mitis-oralis*.

We have previously used this same multiparameter flow cytometric technique to examine HDP-induced killing mechanisms against *S. aureus* [28]. The CM kinetic responses observed in *S. mitis-oralis* during HDP exposure differed substantially from those observed in *S. aureus*. For example, hNP-1 induced PSA expression in *S. aureus* (but not in *S. mitis-oralis*). Further, whereas SDS caused hyperpolarization of *S. mitis-oralis* CM ENR, it caused CM depolarization in *S. aureus*. Likewise, SDS-induced CM PRM was observed in *S. aureus,* but not in *S. mitis-oralis*. In contrast, these two organisms were similar in that neither hNP-1, SDS nor ETOH induced CSP in either. It should also be noted that HDPs from other mammalian contexts appear to have unique mechanisms of action against target organisms, such as *S. aureus*. For example, we previously showed that distinct host defense peptides secreted from mammalian platelets differed in their capacity to depolarize and/or permeabilize *S. aureus* cells [28,39]. Therefore, taken together, the dynamic responses of *S. mitis-oralis* strains to diverse cationic peptides are quite distinct from those in *S. aureus*.

Of special importance, use of this dynamic, multi-parameter flow cytometric fingerprint method enabled three significant new insights: (i) survival adaptations of bacterial pathogens to cationic peptides such as DAP or HDPs differ from organism-to-organism (e.g., staphylococci vs. streptococci), as well as among peptides (e.g., DAP vs. hNP-1 vs. LL-37). (ii) The mechanisms of DAP or HDP killing or resistance in bacterial pathogens are dynamic and multi-modal, rather than static and single-target. This view differs from traditional hypotheses in which these microbicidal effects have been assumed to strictly involve perturbation of CM permeability via a non-specific ‘detergent’ mechanism [39]. (iii) Current findings demonstrate that mechanisms of resistance to DAP and HDPs involve highly coordinated and adaptive responses that change over time of exposure.

We recognize there are several limitations to the current studies: (i) analyzing the effects of DAP and HDPs in vitro cannot fully recapitulate the complex conditions in which they function in vivo, (ii) only pre-specified, selected mechanistic parameters were studied, there are likely mechanisms in addition to those studied here that contribute to resistance to DAP and/or HDP killing, (iii) although previously well-characterized, only one representative DAP-S/DAP-R strain-pair was evaluated and (iv) a limited cadre of HDPs were assessed. It is likely, as noted above, that distinct DAP-R adaptive mechanisms will be disclosed when additional HDPs are studied.
